# The 3D Printing of Calcium Phosphate with K-Carrageenan under Conditions Permitting the Incorporation of Biological Components—A Method

**DOI:** 10.3390/jfb9040057

**Published:** 2018-10-17

**Authors:** Cindy Kelder, Astrid Diana Bakker, Jenneke Klein-Nulend, Daniël Wismeijer

**Affiliations:** 1 Department of Oral Implantology and Prosthetic Dentistry, Academic Centre for Dentistry Amsterdam (ACTA), University of Amsterdam and Vrije Universiteit Amsterdam, Gustav Mahlerlaan 3004, 1081 LA Amsterdam, The Netherlands; c.l.kelder@acta.nl (C.K.); d.wismeijer@acta.nl (D.W.); 2 Department of Oral Cell Biology, Academic Centre for Dentistry Amsterdam (ACTA), University of Amsterdam and Vrije Universiteit Amsterdam, Amsterdam Movement Sciences, Gustav Mahlerlaan 3004, 1081 LA Amsterdam, The Netherlands; j.kleinnulend@acta.nl

**Keywords:** three-dimensional-printing, bioactive bone substitute, biological factor, growth factor, K-carrageenan, extrusion-based 3D-printing

## Abstract

Critical-size bone defects are a common clinical problem. The golden standard to treat these defects is autologous bone grafting. Besides the limitations of availability and co-morbidity, autografts have to be manually adapted to fit in the defect, which might result in a sub-optimal fit and impaired healing. Scaffolds with precise dimensions can be created using 3-dimensional (3D) printing, enabling the production of patient-specific, ‘tailor-made’ bone substitutes with an exact fit. Calcium phosphate (CaP) is a popular material for bone tissue engineering due to its biocompatibility, osteoconductivity, and biodegradable properties. To enhance bone formation, a bioactive 3D-printed CaP scaffold can be created by combining the printed CaP scaffold with biological components such as growth factors and cytokines, e.g., vascular endothelial growth factor (VEGF), bone morphogenetic protein-2 (BMP-2), and interleukin-6 (IL-6). However, the 3D-printing of CaP with a biological component is challenging since production techniques often use high temperatures or aggressive chemicals, which hinders/inactivates the bioactivity of the incorporated biological components. Therefore, in our laboratory, we routinely perform extrusion-based 3D-printing with a biological binder at room temperature to create porous scaffolds for bone healing. In this method paper, we describe in detail a 3D-printing procedure for CaP paste with K-carrageenan as a biological binder.

## 1. Introduction

Critical size bone defects caused by trauma, bone cancer, congenital defects, and overloading are a common clinical problem for which the standard treatment is autologous bone grafts [1,2]. These grafts are used in dentistry, oral and maxillofacial surgery, and orthopaedic surgery to stimulate the formation of new bone. However, current treatment options have significant limitations, i.e., autologous bone grafts are only available in limited volume, and a second surgery is needed for the harvesting procedure which is often associated with co-morbidity [3]. Importantly, autografts have to be manually adapted to fit the defect area. Unfortunately, the achieved fit is not precise, leaving lacunae in the contact area between the autograft and the defect border, which may impair bone healing and prolong the healing period. 3D-printing offers a solution to this problem by enabling the reproducible production of clinically relevant scaffolds of precise dimensions, tailor-made to fit the defect, thereby facilitating bone regeneration.

CaP in scaffolds is often used for bone regeneration since the crystals in CaP-containing scaffolds resemble the hydroxyapatite in natural bone, and the scaffolds are biocompatible. In addition, most forms of CaP dissolve chemically or are resorbed by osteoclasts in the human body, rendering the scaffolds bioresorbable [4]. Subsequently, the CaP can re-precipitate thereby transforming the scaffold surface, which stimulates the formation of bone [5,6].

3D-printing is a term which describes techniques that create objects in a layered fashion. Additive manufacturing techniques used in the medical field today include SLA, selective laser sintering, selective laser melting, and extrusion-based printing. 3D-printing is based on computer-aided planning and 3D-design by using digital modelling and imaging technology (i.e., computer-aided design, or CAD). Therefore, shapes, geometries, and internal structures can be precisely defined. In addition, this method allows for the layer-by-layer build-up of the construct with proteins, drugs, and/or peptides, enabling localized characteristics to fit explicit functions of the construct at a specific site.

Several 3D-printing techniques can be used to fabricate CaP scaffolds. The two major types of printing used to print CaP are powder printing and extrusion-based printing. In extrusion-based printing, materials are extruded from a nozzle. This technique is gaining more and more interest as it can be carried out at room temperature and allows for the incorporation of biological components and/or cells [7,8,9,10]. Extrusion-based printing of CaP is often based on the combination of particles with a polymer. For example, hydroxyapatite particles can be combined with PCL and polylactic-co-glycolic acid (PLGA) [11]. In powder printing, a layer is created by binding particles in a powder bed, either by sintering with a laser (SLS) or binding by drops of liquid (3D-powder printing) [12,13]. Both SLS and 3D-powder printing are used to bind CaP particles together, thereby creating porous and interconnected scaffolds. Unfortunately, these types of scaffolds are often not very strong, and need additional treatment, such as sintering or a chemical treatment, to improve the strength of the scaffold [12,14].

Most CaP scaffolds have very good osteoconductive properties, i.e., they allow the bone to grow along their surfaces. To speed up the bone healing process, the scaffold should actively stimulate de novo bone formation and vascularization throughout the defect. Active stimulation can be achieved by adding biological components, such as bone morphogenetic protein-2 (BMP-2) and vascular endothelial growth factor (VEGF), to scaffolds to enhance vascularization and bone formation [15,16]. The prime method to add biological components to a scaffold is adhesion to the scaffold’s surface. However, this method results in a burst release of the biomolecules in the first hours after incorporation into a defect site [17]. Such a burst release is often disadvantageous since high levels of biological factors are released following an uncontrolled release pattern during a short period of time, thereby possibly causing complications. A solution to this problem might be provided by the incorporation of components into the scaffold, resulting in a more sustained release pattern [18].

The printing of CaP for drug and growth factor delivery is challenging. Techniques using high temperatures or aggressive chemicals, such as SLS and 3D-powder printing, hinder the bioactivity of the incorporated biological components since cells and proteins that are active at body temperature lose their bioactivity at these high temperatures or are denatured by the chemicals. Extrusion-based printing is an option for achieving the incorporation of active growth factors, as this type of printing can operate at room temperature and use biological binders such as collagen and carrageenan. Carrageenan is derived from red seaweed and resembles the glycosaminoglycans (GAGs) in the extracellular matrix [19]. Other desirable properties are the interconnected porous network, elastic behaviour, and the ability to encapsulate cells [20,21]. The 3D-printing of CaP allows the incorporation of the biological component in the CaP and/or in the biological binder when it is performed at room temperature. Although several studies show printing methods for CaP at mild conditions [11,22,23,24,25], only a few studies show the incorporation of biological components such as growth factors [26,27]. Therefore, in our laboratory, we have developed a method to print CaP at room temperature, permitting the incorporation of biologically active factors. The aim of the current methods paper is to describe a method to perform 3D-printing with CaP paste combined with the biological binder K-carrageenan under mild conditions in sufficient detail to serve as a basis for experiments by other laboratories.

## 2. Material and Methods

### 2.1. Production of Calcium Phosphate Paste

The CaP paste is produced by a precipitation reaction according to a modified version of a coating protocol described earlier [18,28]. In brief, a supersaturated CaP solution containing 684 mM NaCl (31434-1KG-M, Sigma-Aldrich, St Louis, MO, USA), 20 mM CaCl_2_·2H_2_O (102382, Merck Millipore, Darmstadt, Germany), 10.1 mM Na_2_HPO_4_·2H_2_O (106580, Merck Millipore, Darmstadt, Germany), and 200 mM HCl (100317, Merck Millipore, Darmstadt, Germany) is made.

**Figure 1:**
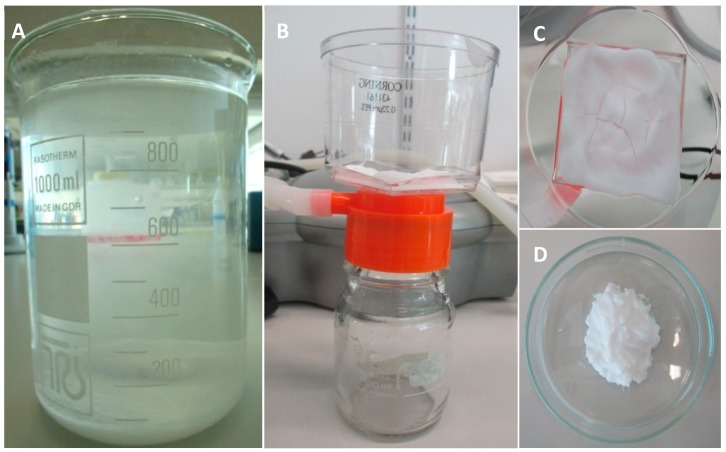
The calcium phosphate precipitation and calcium phosphate paste preparation. (**A**) CaP precipitate after overnight incubation; (**B**) Remaining moisture is removed from the CaP precipitate with a bottle top filter; (**C**) Cracks are formed in the CaP paste, indicating that water removal is sufficient to allow for printing; (**D**) CaP paste is ready to be used for 3D-printing.

The pH is increased from approximately 0.83 to 7.4 (range: 7.35–7.45) by adding TRIS (252859-500G, Sigma-Aldrich, St Louis, MO, USA). After adjusting the pH of the solution, incubation is performed overnight in a shaking water bath at 37 °C to allow CaP precipitation. After incubation, the precipitate (Figure 1A) is retrieved by removing (careful pouring) as much as possible of the supernatant. This precipitate is poured into sterile 50 mL tubes and centrifuged at 1000× *g* for 10 min. The supernatant is removed and discarded. Then PBS (14200-067, Thermo Fisher Scientific, Waltham, MA, USA) is added and the solution is mixed by shaking to wash the precipitate. After five repetitions of washing, centrifuging, and pouring off the supernatant, the last supernatant is removed. The result is a layer of CaP precipitate, from which the remaining moisture is removed by filtering with a 0.22 µL PES bottle top filter (431161, Corning Incorporated, Corning, New York, NY, USA) and an air pump until cracks appear in the CaP (Figure 1B,C). Before water removal with the filter, the CaP ‘slurry’ obtained from 1 L CaP solution is approximately 13.87 g. After water removal, approximately 7.52 g of CaP paste remains (Figure 1D). After preparation, the CaP paste is stored in a syringe with a cap at room temperature. It is advised to use the paste within one week, as water evaporation will occur, thereby significantly influencing the structure of the paste.

### 2.2. Mixing

To print the CaP (with/without biological factors), a Κ-carrageenan (C1804, TCI, Tokyo, Japan) solution with a K-carrageenan concentration of 2.8% (wt/vol; kindly provided by Prof. S. Matsukawa, Tokyo) is mixed with the earlier prepared CaP paste in a 1:1.9 (wt/vol) ratio. The K-carrageenan solution is weighed and heated to 37 °C in a water bath. The amount of CaP paste is determined with a 5 or 10 mL syringe and added to the K-carrageenan in a 7 mL plastic container. To ensure a homogeneous mixture, sonication is performed using a sonicator (Vibra cell™, VCX 400, Sonics, Newtown, CT, USA) with the following settings: amplitude: 20%; pulse: alternating 1.0 s ‘on’ and 1.0 s ‘off’ for 90 s. The mixture is then loaded into a printing cartridge, and ready to be printed.

### 2.3. Incorporation and Release of Biological Factors

To show the incorporation of biological factors in our scaffolds, bovine serum albumin, fluorescein conjugate (BSA-FITC; Molecular Probes, Eugene, OR, USA) is used as a model biological factor. To incorporate the model factor in the CaP paste, the BSA-FITC is added to the precipitate after the washing steps. A total of 100 µL of a 10 mg/mL BSA-FITC solution is added to approximately 10 mL precipitate and mixed with a spatula. Thereafter the standard protocol can be followed by drying the precipitate. To incorporate BSA-FITC in K-carrageenan, 10 µL of 10 mg/mL BSA-FITC is added to the warm (37 °C) K-carrageenan solution and mixed with a spatula. The standard protocol can be followed by adding the appropriate amount of CaP paste and mixing it with sonication. After 3D-Printing, images are made with a Leica fluorescence microscope (Leica Microsystems, Wetzlar, Germany). For each condition (BSA-FITC in CaP paste, BSA-FITC in K-carrageenan and control without BSA-FITC), a scaffold is placed in 2 mL of PBS to measure the release of BSA-FITC from the scaffold. Per condition, the release from 3 scaffolds is measured. At day 3, a 200 µL sample is taken and assayed, in duplicate, for fluorescence intensity at 520 nm with a Synergy HT^®^ spectrophotometer (BioTek Instruments, Winooski, VT, USA).

### 2.4. Three-Dimensional Printing

For 3D-printing, a pneumatic-driven printing head of a 3D-Discovery printer (RegenHU, Fribourg, Switzerland) is used. Before placing the printing cartridge in the printhead, a 23 G (inner diameter: 0.33 mm) blunt needle (Cellink^®^, Gothenburg, Sweden) is attached to the cartridge. Scaffolds are designed using BioCad software (RegenHU, Fribourg, Switzerland), and a circular grid design with a diameter of 15 mm is used. Optimization of the system has resulted in the determination of optimal printing parameters as listed in Table 1. After scaffold printing, the K-carrageenan in the scaffold is cross-linked with an excess of 1 M KCl (104936, Merck Millipore, Darmstadt, Germany) for 30 min.

### 2.5. Scanning Electron Microscope Imaging

For scanning electron microscope (SEM) imaging, samples are fixated overnight with a solution containing 4% paraformaldehyde, 1% glutaraldehyde and 0.1 M sodium cacodylate. Samples are washed with distilled water, dehydrated with ethanol series and dried overnight at room temperature. Samples are coated with gold using an Edwards Sputter Coater S150B (Edwards, Burgess Hill, England) and the images are made with a Zeiss EVO LS-15 scanning electron microscope (Zeiss, Oberkochen, Germany) with an accelerating voltage of 7 kV.

## 3. Results and Discussion

3D-printing is used with increasing frequency to create patient-specific constructs based on computer-aided planning and 3D-design by using digital modelling and imaging technology. In this method paper, we describe a protocol for the printing of CaP at room temperature with the possibility of incorporating biological components.

The first step of the process entails the production of the CaP paste. We tested whether the sequence in which the salts are dissolved during the CaP production process affects the precipitation process. This appeared not to be the case. To estimate CaP mineral maturation in the precipitate, the Ca/P ratio was analyzed. The CaP ratio in 3 samples is 1.46 ± 0.1 (mean ± SD). In the study by Leeuwenburgh et al. (2001), different layers are precipitated as coatings. One of these coatings uses the same salts in the supersaturated solution as we do in our study, and is described as an octacalcium phosphate coating [29]. In our protocol, higher ion concentrations are used, i.e., 5 times higher Na^+^ and Cl^−^ ion concentration, 5.4 times higher HPO_4_^2−^ ion concentration, and 6.5 times higher Ca^2+^ concentration. The Ca/P ratio in our study is slightly higher compared to the Ca/P ratio of octacalcium phosphate, indicating the presence of calcium-deficient hydroxyapatite [30].

**Figure 2:**
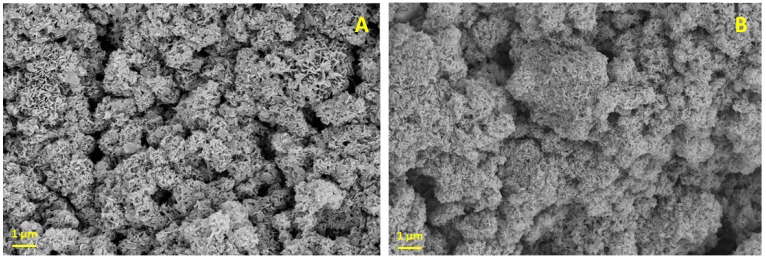
Scanning electron microscopy. (**A**) CaP; (**B**) CaP with K-carrageenan. Magnification: 5000×, Scalebar: 1 µm.

To characterize the CaP material, we used a scanning electron microscope (SEM). Under SEM, dried CaP particles appear as irregular microspheres with plate-like crystals (Figure 2A). When the K-carrageenan is mixed with the CaP paste and dried (Figure 2B), fewer plate-like crystals are present. The exact crystal structure of the CaP after printing with K-carrageenan remains to be determined, but the crystal structure is likely amorphous [31]. We use CaP paste rather than dry particles for printing and do not dry the 3D-printed scaffolds after printing, as this causes excessive shrinking and possibly structural damage to the constructs. From 1 L ion solution, 7.5 g of CaP paste is produced. When all the water is evaporated from 7.5 g paste, approximately 1.4 g of CaP particles is obtained. The paste thus contains approximately 4.4 g of water per gram of CaP precipitate.

Here K-carrageenan is used as a biological binder. Other materials, e.g., bioink from Cellink^®^ or a silanized hydroxypropylmethylcellulose hydrogel, can be used as well. The bioink from Cellink^®^ is composed of nanofibrillated cellulose and alginate. The nanofibrils are morphologically similar to collagen [32], one of the main components in the bone matrix. The bioink is optimised for extrusion-based printing. Silanized hydroxypropylmethylcellulose hydrogels can be mixed with BCP granules [33] and, therefore, can be mixed with CaP. Moussa et al. (2017) show that adipose stem cells are viable inside these hydrogels [34]. We use K-carrageenan because of its resemblance to native glycosaminoglycans and proven biocompatibility [35,36].

**Figure 3:**
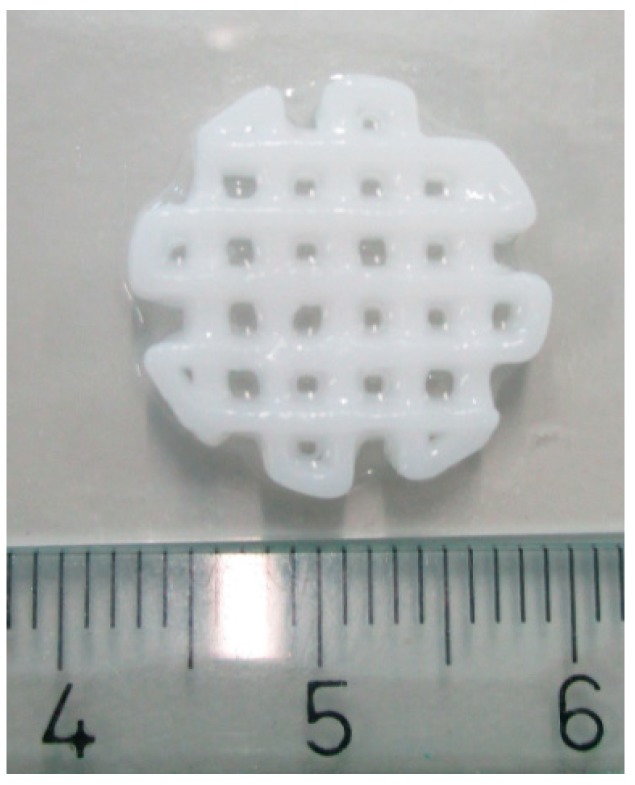
The 3D printed scaffold. Scale in centimeters.

We routinely print scaffolds with a diameter of 1.5 cm and strands of 1–1.5 mm in width (Figure 3). Scaffolds printed in consecutive order vary somewhat in dimensions. We measured the strand width of 3 scaffolds printed in one experiment. The width of the strands varied from 0.8 to 1.6 mm. The average strand width (measured at 10 different points: 5 vertical and 5 horizontal) of the 3 scaffolds was respectively 1.3, 1.2, and 1.0 mm. The average pore size was also measured, which was respectively 0.31, 0.39, and 0.55 mm^2^. In our experience, scaffolds printed on consecutive days had the same dimensions. The maximum printing volume is 130 mm × 90 mm × 60 mm.

For the printing of viscous composites, optimization steps are needed. For example, the nozzle can penetrate the previously printed layers, which is unfavourable. This problem may be solved by adopting the printing parameters. When the layer thickness (setting of the printer) is increased, the distance the nozzle goes up with every layer increases, thereby solving the problem. Small incremental steps (0.01 mm) are used until the needle stops penetrating the previous layers. In addition, increasing the parameter initial height can also help solve the problem. We tend to print at a pressure of 1 Bar, which creates strands between 1 and 1.5 mm as described above. By decreasing the pressure it is possible to create thinner strands, although this might lead to a disruption of the continuity of the printing process. Another option to decrease the strand thickness is to increase the initial height of the nozzle, as the strands are more stretched while being printed. The advantage of using thinner strands is that it provides increased scaffold porosity, but the disadvantage is that the strands might be mechanically weaker. The thinnest strands that we have obtained using CaP paste and K-carrageenan have a thickness of 0.75 mm.

**Figure 4:**
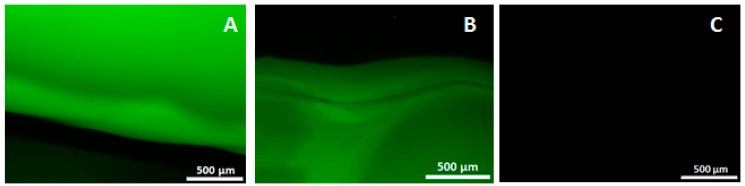
The fluorescent images of incorporated bovine serum albumin, fluorescein conjugate. (**A**) CaP/K-carrageenan scaffold with BSA-FITC incorporated in the CaP paste; (**B**) Scaffold with BSA-FITC incorporated in the K-carrageenan solution; (**C**) Control scaffold without BSA-FITC incorporated. Magnification: 4×.

**Figure 5:**
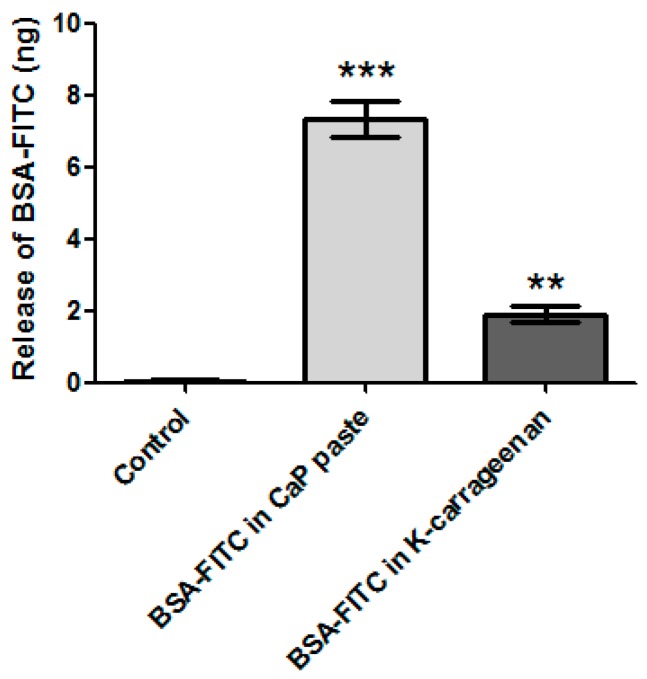
The release of incorporated BSA-FITC. From a CaP scaffold with BSA-FITC incorporated in the CaP paste, a scaffold with BSA-FITC incorporated in the K-carrageenan solution, and a control scaffold without BSA-FITC incorporated. Normalized for the volume of the scaffolds. The significance is compared to the control scaffold, ** is *p* ˂ 0.01 and *** is *p* ˂ 0.001.

The 3D-printing method described in this paper is performed at room temperature and without aggressive solvents, allowing biological components to be incorporated into the CaP and/or in the K-carrageenan. BSA-FITC is used as a model to show the incorporation of biological components in CaP. In Figure 4, a fluorescent signal is visible when BSA-FITC is incorporated into the CaP paste (A), and in the K-carrageenan (B). The materials are not autofluorescent as no fluorescent signal is visible in the control sample (C). The release of BSA-FITC from the scaffolds is measured after 3 days. The cumulative BSA-FITC release from the scaffolds incorporated in the CaP paste and in the K-carrageenan is determined (Figure 5). The BSA-FITC release from K-carrageenan is less than that from CaP paste. This might be explained by the fact that the total amount of BSA-FITC incorporated in CaP paste was 10 times higher than in K-carrageenan. This difference in the amount used was based on the expectation that more BSA-FITC would be lost during its incorporation in CaP paste. We show that biological factors can be incorporated in both CaP paste and in a K-carrageenan solution and that these factors are released from the scaffolds. We estimate that approximately 0.02% of the incorporated BSA-FITC is released from CaP/K-carrageenan scaffolds after 3 days.

Liu et al. (2001) have tested the release of co-precipitated BSA from CaP particles. They found that only 0.3% of the incorporated BSA was released during the first 4 h, which increased to 0.85% after 6 days [18]. It has been reported that the release of BMP-2 increases due to resorption of the material by cells [28]. Our precipitation protocol is based on the protocol of Liu et al. but the release of biological factors from the printed end product is likely to be different from the particles as described by Liu et al. (2001) as we use the precipitated CaP as a paste rather than as dry particles, and we combine the CaP with carrageenan. By experimenting with incorporating biological components in the K-carrageenan and/or in CaP paste, favourable release kinetics can be tailored.

For eventual clinical purposes, it is important that the whole scaffold production process can be performed under sterile conditions. The CaP paste can be produced in a sterile manner since a CaP solution and a TRIS solution can be made separately and filtered with a Millipore filter unit of 0.22 µm. After filtering, the solutions are mixed and incubated overnight in a shaking water bath at 37 °C. The K-carrageenan can be sterilized by heating till 85 °C for 3 h and both the mixing and the 3D printing can be done under sterile conditions, for example, in a clean room.

Taken together, we have provided a detailed protocol for the printing of CaP-carrageenan hybrid scaffold which can be used as a base by other labs to establish their own 3D printing protocol.

## Figures and Tables

**Table 1:** The printing parameters used for the 3D-printing of CaP scaffolds with K-carrageenan as a binder.

Parameter	Value
Initial height (mm)	0.5–0.80 (range)
Thickness (mm)	0.25–0.40 (range)
Speed rate (mm/s)	7
Pressure ^1^ (Bar)	0.5–2.0 (range)
Line space ^2^ (mm)	2.50

^1^ ‘Pressure’ is an adaptable parameter. When printing multiple scaffolds in a row, the pressure might have to be increased; ^2^ ‘Line space’ is the amount of space between the lines of the grid. Line space influences the number of lines used to fill up the grid.
